# Social Stress Increases Cortisol and Hampers Attention in Adolescents with Excess Weight

**DOI:** 10.1371/journal.pone.0123565

**Published:** 2015-04-21

**Authors:** Antonio Verdejo-Garcia, Maria Moreno-Padilla, M. Carmen Garcia-Rios, Francisca Lopez-Torrecillas, Elena Delgado-Rico, Jacqueline Schmidt-Rio-Valle, Maria J. Fernandez-Serrano

**Affiliations:** 1 Department of Clinical Psychology and Institute of Neuroscience F. Oloriz, Universidad de Granada, Granada, Spain; 2 School of Psychological Sciences, Monash University, Melbourne, Australia; 3 School of Health Sciences, Universidad de Granada, Granada, Spain; 4 Department of Psychology, Universidad de Jaen, Jaen, Spain; Centre de Neuroscience Cognitive, FRANCE

## Abstract

**Objective:**

To experimentally examine if adolescents with excess weight are more sensitive to social stress and hence more sensitive to harmful effects of stress in cognition.

**Design and Methods:**

We conducted an experimental study in 84 adolescents aged 12 to 18 years old classified in two groups based on age adjusted Body Mass Index percentile: Normal weight (n=42) and Excess weight (n=42). Both groups were exposed to social stress as induced by the virtual reality version of the Trier Social Stress Task --participants were requested to give a public speech about positive and negative aspects of their personalities in front of a virtual audience. The outcome measures were salivary cortisol levels and performance in cognitive tests before and after the social stressor. Cognitive tests included the CANTAB Rapid Visual Processing Test (measuring attention response latency and discriminability) and the Iowa Gambling Task (measuring decision-making).

**Results:**

Adolescents with excess weight compared to healthy weight controls displayed increased cortisol response and less improvement of attentional performance after the social stressor. Decision-making performance decreased after the social stressor in both groups.

**Conclusion:**

Adolescents who are overweight or obese have increased sensitivity to social stress, which detrimentally impacts attentional skills.

## Introduction

Adolescents with excess weight suffer substantial social stress including frequent peer bullying and social marginalization and exclusion [1,2]. Crucially, the degree of exposure to these social stressors is the most important predictor of poor psychological adjustment and poor academic achievement in adolescents with obesity [3]. Moreover, neuroendocrine studies have shown that non-fasting levels of the “hunger hormone” ghrelin increase in response to social stressors (i.e., the Trier Social Stress Task, involving a public speak) [4] and that the awakening response of the “stress hormone” cortisol positively associates with subsequent lipid intake [5]. Therefore, social stress is a potent determinant of poor cognition and poor food choices in adolescents with excess weight. This phenomenon could be explained by the harmful impact of social stress on cognitive skills such as attention, cognitive control and decision-making, which contribute to obesity-related behaviours in adolescents [6]. The harmful impact of persistent social stressors on cognition in adolescents with obesity is likely to be enduring as stress induces neuroadaptations in prefrontal and limbic regions particularly during adolescence [7,8]. Therefore, examining whether social stress hampers cognition in adolescents with excess weight is essential for prevention of cognitive decline and hence progression of obesity. However, to date no studies have experimentally assessed this notion. In this study we examined if a social stressor-the Trier public speaking stress task- specifically increases cortisol levels and hampers cognitive performance in adolescents with excess weight compared to adolescents with normal weight. We specifically assessed the impact of social stress on outcome measures of attention, cognitive inhibition and decision-making. We selected these outcomes because they reflect the function of frontal-limbic systems [9,10] and are longitudinally associated with weight gain in pediatric populations [11,12]. We hypothesized that adolescents with excess weight would show greater cortisol response to the social stressor, and greater detrimental impact of social stress on attention and decision-making performance.

## Methods

### Participants

Eighty-four adolescents aged between 12 and 18 years old participated in the study. They were classified in two groups (Normal weight [n = 42] and Excess weight [n = 42]) based on their age adjusted Body Mass Index (BMI) percentile [13]. Sample size was estimated through power analysis. The existing evidence about the impact of the Trier Social Stress Task (TSST) on selected outcome variables was correlational (i.e., the association between TSST-induced cortisol changes and decision-making performance is between 0.3 and 0.4) [14,15]. Therefore, we estimated that in order to achieve adequate power (80%) to detect a *ρ*H1 = 0.3 association between the independent variable (stress) and the cognitive outcomes (attention and decision-making) 84 participants would be required (S1 Fig). This sample size was deemed acceptable for the mixed repeated-measures design. The classification of the two groups was conducted in alignment with the guidelines of the International Obesity Task Force and the Centers for Disease Control and Prevention: Normal weight participants had age adjusted BMI percentiles in the range between the 5^th^ and the 84^th^ percentile, and Excess weight participants had age adjusted BMI percentiles ≥85 (Table 1). Three participants from the Excess weight group provided invalid cortisol samples, and therefore the final study sample comprised 42 Normal weight and 39 Excess weight participants. Participants’ socio-demographic characteristics, BMIs, percentage fat and blood count obtained biochemical parameters are as well displayed in Table 1. Participants also completed The Dutch Eating Behavior Questionnaire [16] which was used to characterise psychological traits relevant to maladaptive eating behaviours (i.e., external eating, emotional eating and restraint) (Table 1).

**Table 1:** Descriptive scores for the demographic, biometric and blood count characteristics of adolescents with excess and normal weight.

	Excess weight		Normal weight			
Variables	Mean	SD	Mean	SD	t^a^/chi square^b^	p
Age	15.59	1.91	15.62	1.83	-.07^a^	.944
Gender (% Men/Women)	52.4/47.6		43.2/56.8		.72^b^	.262
BMI	29.87	3.57	20.87	2.06	13.73^a^	.000
Fat (%)	31.97	9.15	17.99	6.94	7.69^a^	.000
DEBQ							
Emotional	23.68	9.33	24.03	9.02	-0.17^a^	.858
External	28.64	7.11	31.28	7.68	-1.71^a^	.091
Restraint	25.55	7.22	19.30	7.81	3.98^a^	.000
Glucose	92.57	6.03	92.14	6.64	.276^a^	.783
Cholesterol	158.07	27.35	148.47	20.89	1,64^a^	.104
Triglycerides	70.64	28.76	63.13	27.32	1.09^a^	.279
HDL	56.80	12.49	58.73	12.82	-.61^a^	.541
LDL	90.85	21.08	80.51	14.96	2.38^a^	.020
Insulin	47.28	57.78	53.34	114.18	-.25^a^	.802
Uric Acid	5.08	0.87	4.39	0.97	2.98^a^	.004
Thyroxine	1.33	0.43	1.44	0.68	-0.79^a^	.451

^a^value of *Student’s t;*

^b^value of *Chi-square* χ^2^

Participants were recruited from the paediatrics and endocrinology services of the Hospital “Virgen de las Nieves” in Granada (Spain), and from schools located in the same geographical area. The inclusion criteria for participants were defined as follows: (i) age range between 12 and 18 years old; (ii) BMI percentiles falling within the intervals categorized as overweight or obesity (≥85—Excess weight group), or normal weight (5–85—Normal weight group); and (iii) absence of history or current evidence of neurological or psychiatric disorders, assessed by participants and parents interviews and the Eating Disorder Inventory [17]. All participants had normal or corrected-to-normal vision.

### Experimental procedures

**Fig 1:**
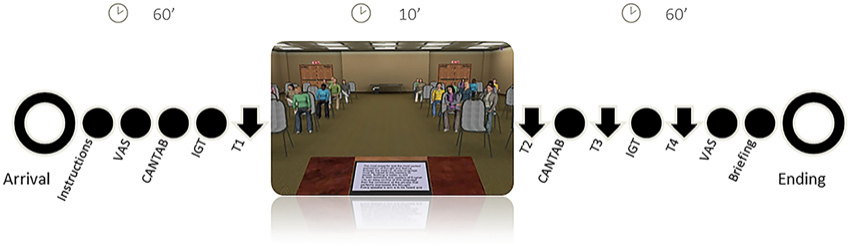
Schematic representation of the experiment.

Fig 1 displays a schematic representation of the experiment. In order to induce social stress in the laboratory we utilised a previously validated Virtual Reality version of the Trier Social Stress Task (TSST) [18]. Participants had to perform a stressing task which consisted of delivering a speech about personal characteristics including both positive and negative aspects of themselves in front of a simulated audience. Participants were told that this audience would attend the speech and subsequently evaluate its quality. However, the virtual audience was programmed to look progressively bored and disappointed with the speech. The speech was followed by a mental calculation test (serially subtracting 17, starting from 2013). Cortisol levels were measured via saliva samples collected before onset of the TSST (T1), after completion of the TSST and the calculation test (10 minutes after TSST onset—T2) and after performance on each of the attention and decision making cognitive probes (20 and 30 minutes after TSST onset-T3 and T4- respectively). Cognitive measures were conducted in a fixed order before TSST onset (pre-TSST, overlapping with T1) and after completion of the TSST and the calculation test (post-TSST, overlapping with T2). To minimize practice effects, we utilised parallel versions of all tasks in the post-TSST administration. The original validation study showed that this virtual reality TSST is able to induce modest but sizeable increases in cortisol and subjective stress responses [18]. Moreover the virtual audience tamed the ethical concerns associated with the negative impact of the social stressor on adolescents’ participants. The Ethics Committee for Human Research of the Universidad de Granada approved the study. Both participants and parents signed informed consent.

#### Cortisol measurement

Participants were told not to smoke, eat or drink coffee for at least 30 minutes before the experiment. All the experimental sessions were conducted at the same time of the day (4–5 pm) based on pilot data obtained in this cohort prior to study onset indicating that diurnal cortisol levels were stable during these hours. Saliva was collected via a commercially available device: Salivette Cortisol (Sarstedt, Numbrecht, Germany). This device is composed of a cotton tube (similar to dental cotton), and two plastic tubes that fit one inside the other. Subjects were told to place cotton salivettes inside their mouth and gently chew and/or suck on them for 1–3 min until they became soaked in saliva. The cotton tube was inserted inside the plastic tube, which was then capped. Saliva samples were stored at -20°C until required for assay. Samples were analyzed at the University Hospital, using the electrochemiluminescence immunoassay “ECLIA” method. This method is designed for use in Roche Elecsys 1010/2010 automated analyzers and in the Elecsys MODULAR NALYTICS E170 module. We computed two different metrics from each cortisol sample (microgram/deciliter and nanomol/liter). The correlation between both metrics at the different time points ranged from 0.8 and 0.9.

#### Cognitive measures

We utilized three computerized tests: two subtests from the Cambridge Neuropsychological Test Automated Battery (CANTAB) [19], *Motor Screening (MOT)* and *Rapid Visual Information Processing (RVP)*, and the Iowa Gambling Task (IGT) [20]. Alternate versions of each test were used in pre-stress and post-stress administrations.

#### MOT

The main objective of this test is to provide a baseline measure of the subjects’ basic motor skills in terms of reaction times and accuracy. After a demonstration of the correct way to point on the computer screen using the forefinger of the dominant hand, the subjects must point to a series of stimuli (crosses) popping up in turn. The outcome measure of this test was response latency.

#### RVP

This is a test of visual sustained attention with an impulse control component. A white box is displayed in the centre of the computer screen, inside which digits, from 2 to 9, are displayed in a pseudo-random order, at the rate of 100 digits per minute. The subject must detect consecutive odd or even sequences of digits (for example, 2-4-6) and respond by pressing the touch pad. The outcome measures of this test were response latency and response discriminability (B’) scores, which are sensitive to attention and impulse control domains respectively. The B’ score is the signal detection measure of the strength of trace required to elicit a response (range -1.00 to +1.00). Thus, it is the tendency to respond regardless of whether the target sequence is present and uses the p(hit) and p(fa) results. A score close to +1.00 indicates that the subject gave few false alarms.

#### IGT

This is a computer task measuring reward/punishment based decision-making. It involves four decks of cards (A, B, C and D). Each time a participant selects a card, a specified amount of play money is awarded. However, interspersed among these rewards, there are probabilistic punishments (monetary losses). Two of the decks of cards (A and B) produce high immediate gains; however, in the long run, they will take more money than they give, and are thus considered disadvantageous. The other two decks (C and D) are considered advantageous, as they result in small, immediate gains, but will yield more money than they take in the long run. The performance measure was the net score calculated by subtracting the number of disadvantageous choices (decks A and B) from the number of advantageous choices (decks C and D). An equivalent parallel version of the ABCD task in which decks are labelled K, L, M and N was utilised in the post-TSST administration. These versions have shown adequate test-retest reliability and ecological validity in relation to decision-making [21].

#### Visual Analogue Scales (VAS)

We used two Visual Analogue Scales (VAS) designed to rate arousal and stress. For arousal scale the individual must indicate the extent to which they perceived as active and alert (from nothing active to very active). For stress scale they must indicate how much stress they feel (from no stress to very much stress). We used the mean scores of each dimension.

### Statistical analyses

The main hypotheses were examined utilizing mixed repeated measures analyses of variance including Time as the repeated-measures factor, Group as the between-groups factor, and cortisol levels (as measured in μg/dl) and RVP’s mean response latency and B’ scores and IGT’s net scores as dependent measures. Cortisol and RVP performance measures were log-transformed (base 10) to meet the normal distribution, but for the sake of clarity the Figures report non-transformed measures. IGT scores fitted to the normal distribution as assessed by Kolgomorov-Smirnov tests. We also performed correlation analyses between change scores of cortisol levels (T2—T1) and change scores of cognitive performance (T2—T1) and between both change scores and biological and psychological measures. These change measures were non-normally distributed and therefore we applied Spearman’s rank correlation analyses. Two participants from the Excess weight group (n = 37) and one participant from the Normal weight group had missing cortisol data at T1 and T2 (n = 41). With regard to cognitive tests, there was no missing data in the Excess weight group (n = 39), whereas in the Normal weight group three participants had invalid data for RVP response latency and IGT (n = 39) at T1 or T2, and three participants had invalid data for RVP B’ (n = 38) at T1 or T2.

## Results

### Cortisol response

**Fig 2:**
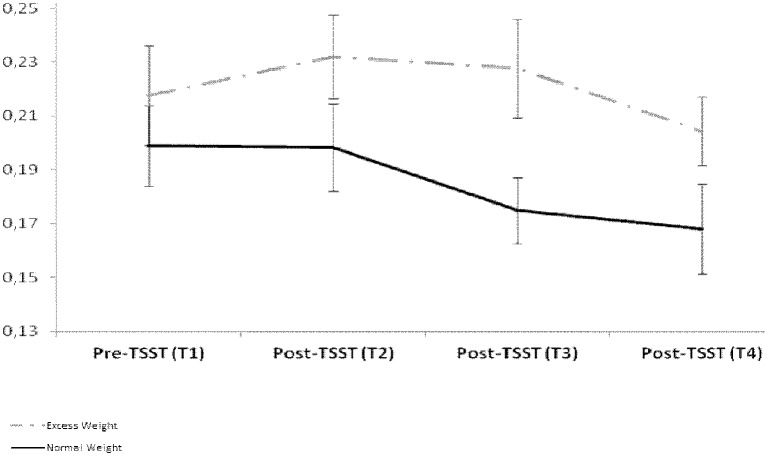
Cortisol levels (μg/dl units) in adolescents with excess weight and adolescents with normal weight before and after exposure to the Trier Social Stress Task (TSST). T1 represents cortisol levels before TSST; T2 represents cortisol levels immediately after TSST termination; T3 and T4 represents cortisol levels 10 and 20 minutes after TSST termination.

We found a significant Time x Group interaction on cortisol levels, F (3,74) = 4.36, p = 0.008. Cortisol mildly increased in Excess weight participants after the TSST. Independent-sample t-tests showed that Excess weight and Normal weight participants did not significantly differ on cortisol levels before TSST (T1). However, Excess weight adolescents showed significantly increased cortisol levels after TSST (T2), t = 1.94, p = 0.05, Cohen’s d = 0.5 (Fig 2). Moreover, cortisol increase between T2 and T1 correlated with amount of fat, Spearman’s Rho = 0.30, p = 0.01. Between-group differences were also statistically significant at T3, t = 2.44, p = 0.02, and T4, t = 2.63, p = 0.01. However, this effect seems to be driven by decreased cortisol levels in the Normal weight group (Fig 2).

### Cognitive performance

#### MOT

**Fig 3:**
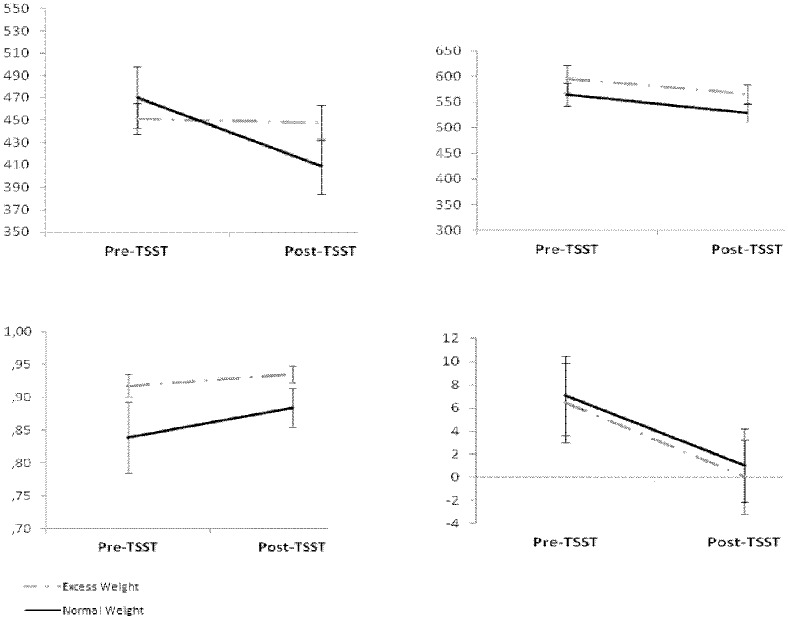
Cognitive performance in adolescents with excess weight and adolescents with normal weight before and after exposure to the Trier Social Stress Task (TSST). Top panel Y axes represent time in milliseconds. The Y axis in the bottom-left panel represents signal detection derived Beta scores, ranging from 0 to 1. The Y axis in the bottom-right panel represents Iowa Gambling Task net scores, ranging from -60 to +60.

Pre-TSST scores showed that both groups had similar baseline response latencies. Further, both groups showed mild reductions of response latencies between the pre-TSST measure and the post-TSST measure (Fig 3).

#### RVP—Response latency

We found a significant Time x Group interaction, F (1,76) = 6.35, p = 0.01 (Fig 3). Independent-sample t-tests showed that Excess weight and Normal weight participants did not significantly differ in the pre-TSST measure. However, they showed marginally significant differences in the post-TSST measure, t (78) = 1.75, p = 0.08, Cohen’s d = 0.4, with Excess weight participants performing significantly poorer than Normal weight controls. There was no significant correlation between T2—T1 cortisol levels and T2—T1 RVP Response Latency.

#### RVP—Response discriminability

We did not find a significant Time x Group interaction, F (1,75) = 0.99, p = 0.32. There were no main effects of Time or Group, although visual inspection shows Excess weight participants performed better than Normal weight participants in both pre- and post-TSST measures (Fig 3).

#### Decision-making—IGT

We did not find a significant Time x Group interaction, F (1,77) = 0.005, p = 0.94. There was a significant main effect of Time, F (1,77) = 6.01, p = 0.02, indicating that both groups exhibited significantly poorer performance after the TSST (Fig 3). There was no significant correlation between T2—T1 cortisol levels and T2—T1 IGT performance.

#### Correlations between biological and psychological measures and cognitive performance in T2—T1

We found a positive correlation between levels of uric acid and change in RVSP response latency performance between T2 and T1, Spearman’s Rho = 0.46, p = 0.0001, and a negative correlation between thyroxine levels and change in Iowa Gambling Task performance between T2 and T1, Spearman’s Rho = -0.27, p = 0.03. We also found a negative correlation between scores of external eating and RVSP response latency performance between T2 and T1, Spearman’s Rho = -0.27, p = 0.02.

#### Visual Analogue Scales (VAS)

We did not find a significant Time x Group interactions on VAS of arousal or stress but results were in the expected direction, with both groups showing more subjective arousal and stress after the TSST (S2 Fig).

#### Post-hoc analyses in the subsample of participants showing enhanced cortisol response

**Fig 4:**
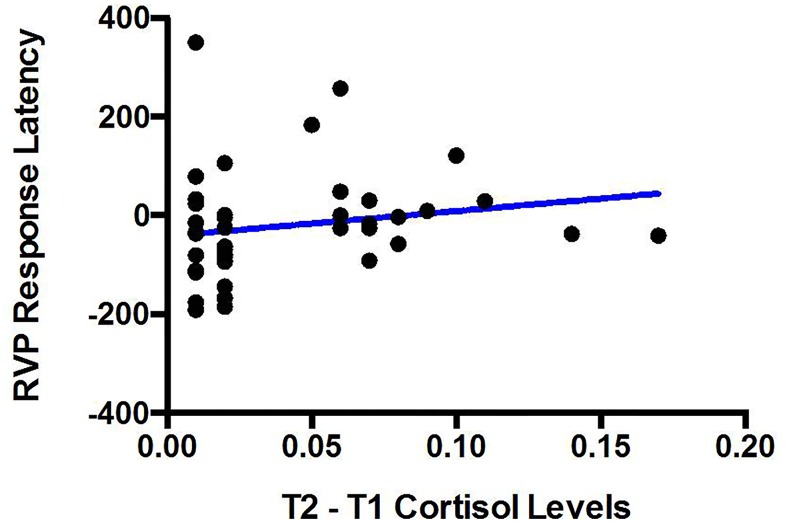
Correlation between between T2—T1 cortisol levels (X Axis) and T2—T1 RVP Response Latency (Y Axis) within the subsample of participants showing TSST-induced increases in cortisol levels.

The primary analyses indicated that in the normal weight group cortisol levels did not change after stress, and therefore there is a concern that cognitive changes were due to spurious factors. To address this issue, we run additional analyses in the subsample of participants who showed sizeable increments in cortisol levels after stress, including 24 participants of the Excess weight group (57% of the original sample) and 20 participants of the Normal weight group (48% of the original sample). The results of these analyses were coherent with the main findings. We found a significant Time x Group interaction on RVP’s latency scores, F (1,41) = 6.17, p = 0.02, whereby a drop in performance was only observed in the Excess weight group (See S2 Fig). Moreover, there was a significant correlation between T2—T1 cortisol levels and T2—T1 RVP Response Latency (Spearman’s Rho = 0.25, p_unilateral_ = 0.05) (Fig 4).

## Discussion

We show that social stress specifically increases cortisol levels and hinders attentional response latency in adolescents with excess weight. Conversely, social stress failed to show significant effects on attention response discriminability. Moreover, both excess weight and normal weight adolescents displayed poorer decision-making performance after the social stressor. These findings indicate that adolescents who are overweight and obese have enhanced stress reactivity in response to social stressors, which selectively impacts on attentional skills. Since adolescents with excess weight are markedly exposed to social stressors during everyday lives, our findings suggest that stress immunization strategies should be put in place to prevent the harmful impact of social stress on cognition and therefore on progression of obesity.

In agreement with our primary hypothesis, social stress induced greater cortisol response in overweight and obese adolescents. The effect was mild but the specific impact on participants with excess weight agrees with the notion that repetitive social stress may induce sensitization of the hypothalamic-pituitary-adrenal (HPA) axis [22] and purportedly of the HPA axis associations with fronto-limbic systems [23–25]. The discrepancy between our finding of cortisol increase and a previous negative finding in obese adults [26] suggests that adolescence compared to adulthood is a more sensitive time period for abnormal sensitization of stress systems, likely due to ongoing neural maturation of these systems [7,27]. Further, both preclinical and clinical evidence shows that social stressors such as social evaluation and social exclusion are particularly challenging for adolescents [8,28,29]. The potential mechanisms for the specific impact of social stress on stress reactivity in adolescents with excess weight include the additive or synergistic interactions between social stress and inflammation [30,31] and/or between social stress and obesity-related neuroadaptations in anterior cingulate and limbic regions that are essential for stress regulation [32,33]. Our finding is particularly relevant in view of the significant association between cortisol reactivity and obesity-related behaviours [34,35], and of the emerging evidence suggesting that high levels of stress can longitudinally predict the progression of obesity [36].

We also showed a significant impact of social stress on attentional performance in adolescents with excess weight. The effect was again mild and pointed to stress-related hindering of the capacity to get benefit from a repeated administration of the task. Previous findings indicate that repeated administration of CANTAB attentional tests is associated with significant improvements in performance (of at least 0.3 in Cohen’s d effect size) [37], and this is what we observed in the control group. However, excess weight adolescents were unable to get benefitted from this repeated administration. The effect was specific for attention-related latency adjustments, but not for psychomotor-related reaction times. Therefore, it suggests a detrimental impact of stress on attention regulation.^6^ This notion is consistent with the neural networks interactions between the HPA axis and medial prefrontal cortex and anterior cingulate cortex regions involved in attention regulation [38–40]. In support, neuroimaging studies have shown that the impact of stress on executive attention is mediated by structural (gray matter) neuroadaptations in prefrontal cortex and anterior cingulate cortex regions [41]. This stress-related attentional hurdle has a high translational value, as individual differences in response latencies to attentional probes are longitudinally associated with increases in BMI [12], implying that adequate control of social stress and/or cognitive boosting of attentional resources may contribute to prevent chronic obesity. This notion is consistent with our finding of significant correlations between less improvement of attentional performance (between T1 and T2) and higher maladaptive eating patterns such as external eating, which reflects attentional bias towards food related cues. Further, both social stress and attentional skills are significantly associated with advantageous social functioning and academic performance [3], and therefore our finding highlights the potential benefit of controlling social stress to improve social and career outcomes in the long-term.

Furthermore, we found poorer decision-making after the social stressor in both adolescents with excess weight and adolescents with normal weight. Since cortisol levels dropped between T3 and T4 (the time window of decision-making task performance) it is unlikely that this finding can be attributed to the effects of acute stress. However, it might be attributed to broader effects of the social stressor, such as the social evaluation context. The latter notion agrees with previous experimental evidence showing that adolescents make riskier choices than young adults or adults when they are under social evaluation [42]. The lack of specificity of our result implies that the impact of social evaluation on decision-making is mediated by neural mechanisms that are similarly sensitized in adolescents regardless of BMI/weight status, or that different neural mechanisms mediate a similar impact of social evaluation on decision-making in excess weight and normal weight adolescents. In favor of the first notion, neuroimaging studies have shown that the impact of social evaluation on decision-making is mediated by increased activation of ventral striatal and orbitofrontal regions [43], which are generally sensitized during adolescence. In favor of the second notion, we have observed that excess weight and normal weight adolescents recruit different brain circuitries during the pondering of social decisions [44]. Future studies are warranted to address this question. In any case, our finding might have general implications for prevention of obesity during adolescence as we know that adolescents who are overweight or obese have higher exposure to social evaluations [3] and that subsequent risky choices are longitudinally associated with weight gain and obesity [11].

We conclude that social stress response is sensitized in adolescents with excess weight, hindering their attentional function. The study has important strengths including the experimental design, the power-informed sample size, the detailed phenotypic characterization and the group matching of excess weight and normal weight adolescents, and the objective measurement of stress reactivity with cortisol biomarkers. However, the results should be as well appraised in light of relevant limitations. It is particularly important to stress that unlike the original TSST [45], the virtual reality TSST was not able to induce significant increases of cortisol levels in the control group. We selected this stressor because it was capable of inducing mild but sizeable stress in the laboratory at the same time that it reduced the ethical implications of stressing “at risk” obese adolescents [18]. In agreement with this assumption, our results indicate that the stress manipulation was actually more effective in obese adolescents (57% of participants showed increased cortisol levels) than in controls (only 48% of participants showed increased cortisol levels). There are however several factors that may explain the variability in stress induction, such as degree of belief in the cover story or degree of immersion in the virtual reality environment, that were not systematically controlled in this study. Therefore, further studies are warranted to reassess the validity of this virtual reality version, and to replicate our findings using TSST versions that are able to unequivocally reproduce the original TSST stress induction. Moreover, in absence of a “no-stress” control condition, we cannot ascertain a causal link between stress and cognitive performance. However, we base our interpretation on previous evidence showing that improvement (rather than stability or decrease) in performance is typically expected in “no-stress” repeated administration designs [37]. A related limitation is the negative finding in relation to cognitive impulsivity. Since mild arousal improves inhibitory control in adolescents, it is plausible that the mild nature of the stressor fostered cognitive impulsivity increases rather than (expected) decreases after TSST. Future studies are warranted to address these limitations, to expand on the biological, psychological and socio-economic mediators of the impact of social stress on cognition, and to longitudinally assess the relevance of this experimental effect on public health indicators of the progression of obesity.

## Supporting Information

S1 Fig Power analysis calculations. (TIF)

S2 Fig Visual Analogue Scales for arousal—left panel- and stress—right panel- in excess and normal weight adolescents before and after the Trier Social Stress Task (TSST). (TIF)
